# What’s new in artificially intelligent joint surgery in China? The minutes of the 2021 IEEE ICRA and literature review

**DOI:** 10.1186/s42836-021-00109-0

**Published:** 2022-01-17

**Authors:** Zhonghua Xu, Yuan Zhang

**Affiliations:** Joint Disease & Sport Medicine Center, Department of Orthopedics, Xinqiao Hospital, Army Medical University, 183 Xinqiao Street, Shapingba District, Chongqing, 400038 China

**Keywords:** Artificial intelligence, Robot, Navigation, Arthroplasty, Domestic product

## Abstract

**Objective:**

To outline the main results of the 2021 International Conference on Robotics and Automation (ICRA 2021) of the Institute of Electrical and Electronics Engineers (IEEE) and review the advances in artificially intelligent joint surgery in China.

**Methods:**

The keynote speeches of the 2021 ICRA were summarized in detail, and publications indexed by five core electronic databases (PubMed, Cochrane, Medline, Embase and CNKI) were systematically surveyed (cutoff date: July 30, 2021) in terms of the main topics of the conference. Publications directly related to artificially intelligent joint surgery in China were identified by using the search strategies of (robotic AND arthroplasty OR replacement), (navigation AND arthroplasty OR replacement), (artificial intelligent AND arthroplasty OR replacement), and (mixed reality AND arthroplasty OR replacement) and systemically reviewed.

**Results:**

While robot-assisted arthroplasty in China is mainly performed using robots made in other countries (*e*.*g*., Mako from Stryker, USA), China’s domestic R&D of robots and clinical studies of robotic joint surgery have made some achievements. Although reports on the safety, effectiveness and clinical efficacy of China’s domestic robot-assisted joint surgery were presented at conferences, they have rarely been published in journals. Existing data indicate that, after the learning curve is overcome, robot-assisted hip and knee replacement surgery can fully achieve the established goals of precision and individualization and can significantly improve the accuracy of prosthesis placement angle and the recovery of the mechanical axis as compared with conventional surgery. The downside is that the low level of intelligentization and individualization means that existing designs are not conducive to personalization during surgery, resulting in low cost-effectiveness.

**Conclusion:**

The safety and efficacy of domestic robot-assisted arthroplasty in China are well documented, and its accuracy and short-term clinical efficacy have been reported. However, the long-term clinical efficacy and the cost-effectiveness of large-scale clinical application of this technique warrants further study. The inadequacies of robot-assisted surgery should be remedied through the deep integration of medicine, engineering and the network.

## Introduction

With the full support of Chinese governments, local and central, artificial intelligence (AI)-assisted joint surgery in China is prepared for a fast track of development. Against this background, the International Conference on Robotics and Automation (ICRA 2021), sponsored by the Institute of Electrical and Electronics Engineers (IEEE), was held in Xi’an in 2021. IEEE is an internationally renowned association of electronic and electrical engineers and is committed to the research and development of electrical and electronic engineering, computer engineering, among others. Currently, IEEE represents one of the most influential not-for-profit technical societies across the globe [1]. It has developed more than 1300 industry standards in the fields of space, computer, telecommunication, biomedicine, power, and consumer electronics, *etc*.

ICRA 2021 was an IEEE-sponsored conference specifically dedicated to the robotics and automation. After the meeting held in China 10 years ago, its return to this country is of special significance. The conference, which featured the theme “AI and Robotics Applications in Intelligent Orthopedics”, was chaired by Professor Kunzheng Wang of Xi’an Jiaotong University and Professors Peifu Tang and Jiying Chen of the General Hospital of People’s Liberation Army (PLA) and was attended by more than 150 surgeons, mechanical engineers, software engineers, network engineers and experts from other disciplines who have been endeavoring to address the hot spots and pain points in the clinical practice of joint surgery by incorporating medicine, engineering and the network.

The conference consisted of four sessions: intelligent orthopedics, robotic arthroplasty, technology enjoyment and debates on pros and cons. In this review, we collated and outlined the keynote speeches and, on the basis of the speeches, we retrieved clinical publications (review papers excluded) published by Chinese investigators over the past 3 years in five core databases (*i*.*e*., PubMed, Cochrane, Medline, Embase and CNKI) to review the advances in AI assistive technology in the field of joint surgery in China.

## Background of and opportunities for intelligent orthopedics in China

Professor Kunzheng Wang delivered a keynote speech titled “AI and the Development of Orthopedics in China”. In fact, AI is also known as “the fourth industrial revolution”, and China’s ability to take the lead in this fourth industrial revolution will dictate the destiny of its orthopedic development in the next several decades. With the advent of precision medicine, computer navigation, three-dimensional (3D) printing and patient-specific instrumentation, the design of new types of joints and the improvement of conventional tools, minimally invasive techniques and robot-assisted surgery have emerged, and they encourage joint surgeons to take new perspectives and set higher goals [2]. Currently, applications of AI in clinical medicine principally involve three main aspects: auxiliary diagnosis, precise positioning and medical robotics. For instance, AI diagnostic models for osteonecrosis of the femoral head, femoral neck fracture and other diseases, established through deep learning algorithms based on X-ray images, allow us to validate the models and adjust parameters using clinical data. In core decompression surgery for avascular necrosis of the femoral head, computer-aided navigation can accurately locate the necrotic area, thereby reducing damage caused by intraoperative fluoroscopy and shortening the operative time. 3D printing enables computer-aided design to move from a virtual space to reality, and its application permits the preoperative simulation of implants in the patient, which substantially improves the precision and safety of joint surgery. What is more, artificial joint design based on computed tomography (CT)/magnetic resonance imaging (MRI) can be individualized with respect to the material, size, shape and biological features of artificial joints. According to Professor Wang, China’s precision medicine program remains in its infancy, and industry guidelines in line with precision medicine plans and the characteristics of Chinese patients needs to be developed to enable precision medicine and joint surgery in China to take a lead in orthopedics by “overtaking on corners”.

Professor Peifu Tang presented a keynote speech on “Applications of the ‘Digital Twin’ Concept in Orthopedics”. The digital twin concept is a reality-transcending concept that enables mapping in the virtual space to reflect the full life cycle of the corresponding physical item through a simulation process involving a multidisciplinary, multiphysical, multiscale and multiprobability integration that makes full use of physical models, sensor updates, operation history and other data. It can be seen as a digital mapping system involving one or more important and interdependent equipment system(s). When applied to orthopedics, it assists in the construction of full-dimensional digital models of “live” human body systems via full-range, cross-scale data perception and digital modeling of the human body for precision and smart medicine. By taking advantage of computer-generated graph and image description techniques, finite element analysis, equivalent mechanics and AI calibration, Dr. Tang’s team conducted a theoretical mechanics analysis and mechanical validation of the three-element stabilization system of the proximal femur. They proposed, for the first time, a machine learning-based fracture classification system upon examining intertrochanteric femoral fracture patterns using the AI clustering algorithm and generated views of anterior, posterior, lateral and medial fractures. They analyzed 504 cases of femoral intertrochanteric fractures and found that most of the fracture lines (anterior view: 85.9%; posterior view: 52.0%; internal lateral view: 49.0%; external lateral view: 62.7%) ran along the intertrochanteric line, which was highly consistent with conventional fracture classification results based on clinical experience, suggesting that the established system is of great significance for describing morphological characteristics and adjuvant therapy [3, 4] (Table 1).

Smart solutions for orthopedic surgery are based mainly on automatic preoperative planning with next-generation AI technology to establish a knowledge- and data-driven method and a systematic, hierarchical and expandable knowledge description system that performs knowledge-aided image processing and preoperative planning and ultimately yields a clear and thorough surgical plan [5]. In recent years, AI and deep learning have flourished in various fields, and the clinical use of robots in joint surgery is a milestone in the development of orthopedics. At this conference, the robot design team presented the process used to design and develop robots for joint surgery. Professor Qinghu Meng from Southern University of Science and Technology first reviewed the development of robots, from industrial robots to conventional medical robots to minimally invasive surgery robots, focusing on wireless capsule endoscopy robots and image-based automatic diagnosis systems, which have a diagnostic accuracy up to 100% [6]. According to Prof. Meng, intelligent medical care will be developed for frequent, real-time and home-based physical examinations, unmanned intelligent care in infectious disease wards and intensive care units (ICU), supermarket- and home-based diagnosis and treatment of digestive diseases, the specialization and miniaturization of robot-assisted surgery, and the digitalization and intelligentization of orthopedic robot-assisted surgery.

The speech given by Professor Fangwen Zhai from the Intelligent Robot Research Center, Institute for Artificial Intelligence, Tsinghua University, was about “The Future of Orthopedic Robots”. He pointed out that most orthopedic surgical robots, such as spinal surgical robots, joint surgical robots and trauma surgical robots, can perform only relatively simple surgical procedures at present. Therefore, multiple sets of surgical robots must be purchased to operate on different body parts, which increases cost, lengthens the learning curve (LC), and consume more surgical resources. More importantly, the lack of standards and sharing mechanisms among industries and the lack of openness or compatibility of software for various robot systems render it impossible for preoperative planning and robot control software to interact with each other and cause serious data segmentation, which seriously prevents the industry from moving forward. Therefore, it is imperative to develop a unified infrastructure and a common software and hardware platform and to work out various functional modules based on that platform to allow compatibility among surgical modes for different surgeries. Surgery 4.0, launched by Johnson & Johnson, is just one of such unified platforms [7].

Professor Zhai pointed out that intraoperative registration is a bottleneck for robotic surgery because of high skill requirements on doctors, the radiation exposure during long-time intraoperative imaging and point registration and surface scanning methods that affect the surgical process. Therefore, it is necessary to develop a highly efficient automatic registration mode that offers clear and radiation-free real-time imaging support and fast registration algorithms coupled with more flexible scene perception methods and to establish a registration-free surgical navigation mode that provides preoperative qualitative planning, intraoperative multimodal real-time perception and real-time surgical plan generation. In addition, he emphasized that, at present, surgical robots are still a high-degree-of-freedom locator, lacking real-time environmental perception and complex decision-making ability and having weak force control. In the future, robot designs need to be customized and specialized to make robots more dexterous, gentler and more adaptive to various types of surgery.

From the perspective of a surgeon, Professor Jiying Chen comprehensively answered the question “What kind of robot is needed for joint surgery?”. He focused on the shortcomings of existing robots for joint surgery, such as the lack of data accumulation, a low level of intelligentization and the need for manual assistance to complete the operation. The high demand for wide-scope preoperative radiography and the implantation of the reference frame in robot-assisted hip and knee surgery are likely to cause damage to the patient. The cumbersome intraoperative registration procedure and high failure rate will prolong the operation time. The inability of robots to perceive soft tissue tension makes them unable to provide forecasting factors of the impact and dislocation. In view of this, Professor Chen proposed a vision for the future of joint surgery robots: a focus on improving the function and effectiveness of robots, increasing their 3D accuracy and enhancing the individualization of force lines, positions and angles; continuously incorporating the latest knowledge into preoperative planning and enriching personalized design, *e*.*g*., by considering the role of the hip-spine relationship in the hip joint and the roles of developmental deformities and extra-articular deformities in the knee joint.

The clinical benefits and ethical risks of robotic joint surgery are an important research direction that cannot be ignored. The speech made by professor Jin Lin from Peking Union Medical College Hospital was titled “Focus on Clinical Value: Reflections on the Innovation of the TKA Robot, a Chinese Domestic Product”. In his keynote speech, he defined the clinical value from six dimensions, *i*.*e*., patient benefits, clinical pain point resolution, doctors’ self-realization, clinical application costs, learning curve (LC), and the payer’s net expenditure. Of these, patient benefits, clinical pain point resolution, and doctors’ self-realization were used as the numerator items, and clinical application costs, doctors’ LC and the payer’s net expenditure as the denominator items. He asserted that if a product can achieve breakthroughs in two of the six dimensions, the product can be called a “great breakthrough”. Furthermore, he stated that the “one-vote veto” should be avoided due to the difficulty in moving forward along certain dimensions. He further pointed out that robotic surgery is a powerful tool for achieving technological breakthroughs and that although it currently has some shortcomings, surgeons should put more trust in the robot-assisted surgery.

## 5G network and mixed reality in orthopedics

China has built up the largest 5G network base stations in the world, and the widespread application of 5G network offers great opportunities for telemedicine. By taking advantage of 5G communication technology, experts at the General Hospital of PLA remotely guided its surgeons at Hainan Branch to complete a robot-assisted total hip arthroplasty (THA) on two patients. During these procedures, the human-robot communication was smooth, the manipulators and software worked stably, and the surgeries achieved satisfactory short-term efficacy. These surgeries combined robotic operation and 5G communication technology, two state-of-the-art technologies, and attained preliminary results that supported the safety and potential value of 5G communication technology in telemedicine. 5G communication technology-based telemedicine can provide technical support for the hierarchical diagnosis and treatment policies, and what the PLA General Hospital did represents a valuable attempt to apply telemedicine into surgical operations [8].

Mixed reality (MR) technology is a brand-new digital holographic imaging technology and a new interdisciplinary frontier [9] that has stemmed from virtual reality (VR) and augmented reality (AR) technologies in the graphics field. The progress in MR in recent years has broken through the limitations of preoperative planning, education and training and has been profoundly integrated into the entire clinical decision-making and implementation processes. Professor Yihe Hu from Xiangya Hospital of Central South University gave a keynote speech on “The Application of MR in Orthopedic Surgery”, in which he proposed a new strategy for real-time automatic registration that combined MR with 3D printing. His team successfully completed the first MR-assisted THA in China, offering valuable experience for the further application of MR in clinical medicine [10]. Professor Hu further pointed out that MR is a technology that combines the physical world and the digital world and enables the user to interact with digital information or holographic images without departing from the physical world. Thereby, it establishes an interactive feedback information loop among the virtual world, the physical world, and the user to enhance the perceived reality of the user experience. In this way, it serves as a key element in real-time interaction among the physical world, the virtual model and the user that can enable a more intuitive 3D understanding of the lesion preoperatively and can provide new intraoperative options for real-time assessment of virtual 3D images of the bones, peripheral nerves and vascular tissues in the diseased area, allowing surgeons to understand the conditions of the operative area in real time manner [10] (Table 1).

The integration of network communication with digital technologies, especially with MR, is another trend in intelligent medicine. The processing, analysis and computation of massive AI data on the MR cloud platform can achieve seamless connections with large teaching hospitals for remote communication, teaching and precise surgical guidance, which can effectively resolve the problem of inadequate equipment and technology in remote and poor regions. In his keynote speech titled “Remote Consultation and Surgery through the 5G MR Cloud Platform”, Professor Zhewei Ye from the Union Hospital of Huazhong University of Science and Technology demonstrated that 5G is capable of smoothly transmitting high-definition audio and video signals without stuttering or delay [11] and predicted that the emergence of “5G + MR” technology will have a revolutionary impact on medical training, knowledge dissemination and education, clinical decision-making and treatment. His team completed the world’s first 5G + MR-assisted remote consultation and screw fixation of a thoracic fracture in July 2019, by using a 5G network (China Telecom). Through the 5G network, remote preoperative discussion, doctor-patient communication and on-site surgical guidance were successfully implemented, and the remote medical consultation was transformed from the conventional two-dimensional presentation (*e*.*g*., video, images) to the new 3D stereoscopic presentation, which significantly improved surgical accuracy and safety [11, 12]. With this model, 5G + MR-assisted joint surgery will also become feasible (Table 1).

## Computer-navigation in orthopedics

Computer navigation is a representative application of AI in precision medicine. Conventional arthroplasty relies on the doctor’s experience and the visual observation of anatomical landmarks, which can lead to misaligned prosthetic implantation. Computer navigation can significantly improve the accuracy of prosthesis implantation and has been widely used in hip and knee replacement surgeries. Guo *et al* [13] used the data of 106 patients to compare the imaging differences for postoperative prostheses between computer navigation-assisted THA (V-Xp, Stryker, USA) and conventional THA. Their results showed that in patients who underwent computer navigation-assisted THA, the acetabular abduction angle was in the range of 30°–54°, with an average of (40.6 ± 5.1)°, and an acetabular abduction angle of > 50° was observed only in 1 case; in patients who received conventional THA, the acetabular abduction angle was in the range of 28°–70°, with an average of (44.2 ± 8.7)°, and an acetabular abduction angle of > 50° was observed in 10 cases, indicating that computer navigation-assisted THA can significantly reduce outliers in the prosthesis implantation angle within the safe range (Table 1).

In a randomized clinical trial on computer navigation-assisted surgery (Vector Vision CT-free Navigation, BrainLab, Germany) and conventional total knee arthroplasty (TKA), Guoqiang Zhang *et al* from the General Hospital of PLA found that although patients who underwent both types of surgeries did not differ significantly in their preoperative Hospital for Special Surgery (HSS) scores (47.6 *vs*. 45.4), they differed significantly in the coronal and sagittal alignments of the prosthetic knee joint: 28% of the patients who received a conventional TKA had a prosthesis implantation deviation greater than 3°, while none of the patients who received computer navigation-assisted TKA had a prosthesis implantation deviation greater than 3°, and the outlier coefficient of the patients who underwent conventional TKA was three times that of the patients who underwent computer navigation-assisted TKA [14] (Table 1).

In this conference, Professor Zhang gave a special ICRA presentation on his pioneering work using the Brainlab KNEE3 navigation system (Smith & Nephew, USA) to precisely guide TKA [15]. He reported that the clear and precise preoperative planning of the system allowed for 3D anatomical reconstruction, and the intraoperative navigation system could monitor changes in various angles during knee joint flexion and extension and internal and external rotations, which is conducive to intraoperative orientation adjustment, emphasizing the operational techniques and key points of medial condylar restoration. The method is important in that the restoration of the anatomical structure and exercise physiology of the medial femoral condyle during TKA is beneficial to the recovery of the tension of the medial joint line and the medial collateral ligament (MCL), thus ensuring stability throughout the surgery (Table 1).

In a clinical study involving 14 female patients with femoral constitutional varus from the southwestern region of China, Yuan Zhang *et al* from Xinqiao Hospital of Army Medicial University demonstrated that computer navigation-assisted TKA (StealthStation S7, Medtronic, USA) could help reduce the release range and operative time during the handling of medial soft tissue structures of the knee with osteoarthritis due to constitutional varus. Of 7 patients who underwent computer navigation-assisted TKA, none had pie-crust releasing of MCL or peeling-off of its attachment on the posteromedial tibia; in 7 patients who received conventional TKA, 4 had releasing of the superficial MCL, and 3 had peeling off of the tibial attachment. The patients who underwent computer navigation-assisted TKA had a soft tissue operative time that was 6.1 min shorter on average than that of the patients who underwent conventional TKA [16].

In another of his study, Zhang *et al* examined the perioperative blood loss of 13 patients who underwent unilateral posterior cruciate ligament retaining TKA with the aforementioned navigation system and found that the total blood loss was marginally but not significantly higher (GROSS method: 61.6 mL; HB-balance method: 41.3 mL) in patients receiving computer navigation-assisted TKA than in their counterparts undergoing conventional TKA. However, the hidden blood loss was significantly lower in patients undergoing computer navigation-assisted TKA than in those receiving conventional TKA [computer navigation-assisted TKA: 448.7 ± 300.7 mL (GROSS method) and 374.3 ± 360.8 mL (HB-balance method) *vs*. conventional TKA: 688.6 ± 405.8 mL (GROSS method) and 667.2 ± 425.5 mL (HB-balance method)] [17]. These results further confirmed that computer navigation has the advantages of precision and minimal invasiveness and can find a wide range of applications in both regular or complex primary TKA (Table 1).

## Robotic surgery in hip arthroplasty

Currently, the clinical efficacy of surgical robots represents a hot topic among joint surgeons. At present, the Mako robot (Stryker, USA) is undoubtedly the most successful among all robots designed for THA. Some Chinese investigators have conducted clinical research on robot-assisted THA. In a clinical study including 118 cases of hip dysplasia, the accuracy rates for implanting the acetabular cup into the Lewinnek and Callanan safety zones were 94.9 and 74.6%, respectively, with Mako robot-assisted THA and 79.7 and 50.8%, respectively, with conventional THA. The bleeding volume and operative time was not higher in patients undergoing robot-assisted THA than in those receiving conventional THA [18]. Li *et al* [19] reported that the postoperative leg length discrepancies (LLD) of patients who received Mako robot-assisted THA (54 hips) and those who underwent conventional THA (54 hips) were 2.3 mm and 6.7 mm, respectively, and the patients with absolute postoperative LLD exceeding 5 mm accounted for 2.6% in Mako robot-assisted THA group and 47.3% in the conventional THA group, indicating that the Mako robot can provide a solution to the inability of conventional THA to achieve equal leg length at the lateral position (Table 1).

In his ICRA keynote speech titled “Preliminary Implementation and Results of a Hip Joint Robot”, Professor Xiaogang Zhang from Xinjiang Medical University noted that robot-assisted artificial hip joint surgery consists of three steps: preoperative virtual planning, the integration of virtual imaging and reality and performance of the actual operation against the virtual plan. Robot-assisted THA can significantly improve the precision of prosthesis placement and has obvious advantages over conventional THA in the restoration of the full lengths and rotation centers of both lower limbs. He focused on the complications related to Make robot-assisted THA and reported that, among 64 cases, 2 experienced loosening of the iliac assembly that made registration impossible and required switching back to conventional THA; 1 case developed iliac bone fracture; 3 cases had errors in acetabulum registration that led to the misalignment of the acetabular cup due to anteversion; and 1 case showed acetabular cup loosening. These complications suggest that the full adoption of virtual preoperative planning still carries some risks during the actual operation (Table 1).

The above study represents an effort made by orthopedists to integrate robots into conventional primary THA. However, the value of robots in complex primary THA has been hardly investigated. In a cohort study, Chai *et al* [20] compared 35 cases of Mako robot-assisted hip joint fusion with 37 cases of conventional primary THA and found that the percentage of cases with the acetabular rotation center in the safety zone in the Mako robot-assisted hip joint fusion group was 94.29%, which was significantly higher than that of the conventional primary THA group (67.56%). In addition, there were significant differences in the left and right anteversion angles of patients in the conventional THA group, which were 21.14° and 16.00°, respectively (*P* = 0.042), while robot-assisted THA could effectively eliminate the deviations in anteversion angles caused by manual operation.

In another patient cohort that included 1 case of hip dysplasia, 1 case of ankylosing spondylitis and hip fusion and 1 case of post-traumatic hip osteoarthritis, Chai *et al* followed-up the patients who received robot-assisted complex THA. The operations went uneventfully, with no complications taking place. The postoperative Harris scores were 83/86 (left/right) for hip dysplasia, 87 for post-traumatic hip osteoarthritis and 62 (poor) for ankylosing spondylitis, suggesting that robot-assisted surgery was suitable for complex THA, especially when the bony landmarks were absent [21] (Table 1).

Professor Wei Chai also gave a special ICRA presentation on “Computer-assisted THA for Spine-pelvic Dysfunction” and noted that the post-THA risk of dislocation and revision was higher in patients with spinal fusion than in patients with normal spinal function (dislocation within 5 years: HR = 1.67; revision within 5 years: HR = 1.53) [22]. The normal hip-spine alignment allows for coordinated motion through the interaction among the spine, pelvis and hip, which jointly maintain the coronal and sagittal balance of the human body [23]. The hip-spine alignment might be disrupted or lost in some THA patients with dislocation for unknown causes. To address this difficult clinical problem, Dr. Chai’s team developed an improved calculation technique for computing safety zone for the Mako surgical robot system that draws the safety zone of the acetabular cup as the patient performs three motions: flexion and 45° inward rotation, straightening and outward rotation and deep flexion. This algorithm also includes some adjustment parameters, sets the acetabular angle with the supine pelvis serving as the reference, uses the acetabular angle corresponding to the erect pelvis to determine whether the straightening external rotation motion is safe and employs the acetabular angle corresponding to the pelvis in the sitting position to determine whether hip flexion is stable to provide accurate guidance for acetabular cup implantation. Since no effective treatment is available for spine-pelvis dysfunction in clinical practice, the study by Chai *et al* is significant because it provides viable options for addressing this problem.

Although primary arthroplasty is currently the main field where surgical robots are being used, some Chinese scholars have been aggressively exploring the application of these robots in joint revision surgery. Professor Yixin Zhou *et al* [24] of the Beijing Jishuitan Hospital presented his ICRA report on “Applications of Robots in Complex Hip Revision”. They employed the Mako robot to perform hip revision on 47 patients with failed THA hospitalized from October 2019 to December 2020. All patients underwent whole-body imaging evaluation in the standing and sitting positions preoperatively. On the basis of the evaluation, the Mako robot system calculated the prosthetic safety zone of each patient. Intraoperatively, all patients were successfully registered, and satisfactory clinical outcomes were attained (average operative time: 176 min; average intraoperative blood loss: 885.8 mL; average abduction angle of the acetabular cup: 41.94°; average anteversion angle: 13.94°). Among 45 cases of acetabular revision, the acetabular cup was within the Lewinnek safety zone in 43 cases and within the Callanan safety zone in 38 cases. The results indicated that intraoperative registration is the core element of robot-assisted complex revision of the hip joint. Of the 45 acetabular revision cases, registration was achieved through the bone bed in 27 cases, through the lining in 10 cases, through the acetabular cup in 7 cases and through the cage in 1 case. The power of this study lies in the fact that it has broken through the registration bottleneck of robot-assisted surgery for acetabular revision (Table 1).

## Robotic surgery in knee arthroplasty

Robots play an equally important role in knee arthroplasty. Robot-assisted knee arthroplasty has been successfully extended to unicompartmental knee arthroplasty (UKA) and TKA in China. Fu* et al* [25] examined the learning cureve (LC) in 8 cases of Mako robot-assisted UKA and showed that, during the LC, Mako robot-assisted UKA took a longer operative time than conventional Oxford UKA (131.3 min *vs*. 100.4 min) but had fewer postoperative errors related to the implantation orientation of the prosthesis. In his ICRA report regarding Mako robot-assisted UKA, Professor Qi Wang of the Shanghai Sixth People’s Hospital listed the problems of conventional UKA during bone cut, prosthesis installation and problems with soft tissue balance, *etc*. A long-term follow-up study showed that the 15-year prosthesis revision rate of conventional Oxford UKA was as high as 18.2% [26]. Professor Wang further emphasized that a good balance between soft tissue and trajectory can be accomplished through precise preoperative planning, determination of the intraoperative cutting amount (by measuring the femoral and tibial cortical bone thickness) and intraoperative fine-tuning of the operation plan so that the l-to-2-mm laxity in medial and lateral sides allows for good prosthesis alignment and soft tissue balance during the flexion and extension of knee joint. Furthermore, the Mako robot-assisted UKA can achieve good knee function and excellent short-term postoperative efficacy and offers a good subjective experience for the patient [27], (Table 1).

Professor Jin Lin from Peking Union Medical College Hospital performed 24 TKA by using China’s first domestically-developed surgical robot (HURWA, Beijing Hehuaruibo Medical Technology) [28]. The clinical study showed that, compared with the control method, robot-assisted TKA took a significantly longer operative time (robot-assisted TKA: 114 ± 20 min; control method: 81 ± 11 min; *P* = 0.02) but had insignificantly different postoperative HSS scores (robot-assisted TKA: 84.6; control method: 84.7 points) and WOMAC scores (robot-assisted TKA: 59.6; control method: 63.6). Using a postoperative hip-knee-ankle angle of less than 3° as the eligibility standard for mechanical force line correction, the passing rates were 63.6% for robot-assisted TKA and 69.2% for the control method (*P* = 0.462). This finding confirmed that the HURWA robot is safe, although the robot did not show any advantage over the control method in terms of the lower limb alignment (Table 1).

In his report titled “R&D and the Experience with a Knee Replacement Surgical Robot”, Professor Huiwu Li from the Ninth Hospital of Shanghai Jiaotong University shared his experience with the independent development of the Honghu surgical robot (Honghu, Microport, China). Specifically, during preoperative planning, the graph theory-based joint model automatic segmentation algorithm, physical feature points-based prosthesis matching and planning positioning algorithm were innovatively used to ensure that the CT segmentation quantification indexes of the femur and tibia reached 96.52 and 95.69%, respectively. In addition, the anatomical landmark calculation constraint algorithm was introduced to reduce the registration error to within a range of displacement ≤1 mm and a rotation angle ≤1°, which solved the technical problem of the low speed and poor accuracy of the conventional point cloud registration algorithm. The gravity compensation algorithm was introduced to address the problem of osteotomy inaccuracy due to the large swing of the robotic arm. The use of this algorithm, in combination with optical navigation techniques, achieved real-time precision compensation that was able to control the osteotomy positioning error to within 1.5 mm. An integrated bone-cutting tool was designed that could be applied to all parts of the femoral and tibial bones, thus improving osteotomy efficiency [29, 30] (Table 1).

To best of our knowledge, the largest clinical study in China was performed with Yuanhua (Yuanhua Technology, Shenzhen), another domestic surgical robot. Chai *et al* [31] systematically evaluated robotic arm-assisted bone cut using this system in six goat models and found that the deviation between the planned cutting thickness before operation and the measured value after the operation was < 1 mm and that the error of the osteotomy angle was < 2°, both of which were statistically insignificant (*P* > 0.05). They [32] also investigated the osteotomy angle of this system on six adult cadavers and found that the postoperatively measured hip-knee-ankle angle was 177.1°–179.7°, the coronal femoral component angle was 87.9°–91.4°, and coronal tibial component angle was 87.3°–91.4°, with errors being within ±3° of the angles planned before operation, indicating that this system can assist in precision bone cut based on preoperative planning. After this clinical study, multiple randomized controlled clinical trials involving 180 patients at six joints centers in China and attained encouraging results.

Professor Zongke Zhou from the West China Hospital of Sichuan University presented the results of a clinical trial on Yuanhua robot-assisted full TKA that prospectively enrolled 60 patients with unilateral knee osteoarthritis from October 2020 to February 2021. The cohort included 32 cases in the control group, which underwent conventional surgery, and 28 cases in the test group, which received robot-assisted surgery. Their preliminary results showed that the postoperative lower limb axis deviation ranges were 0–2° for the test group and 0–5° for the control group, with 89% of the patients in the test group having a difference within ±2 mm between the medial and lateral gaps in 90 degrees of flexion, and a difference within ±2 mm between the medial and lateral sides in full extension. The posterior slopes of the tibial component were 2.8° in the test group and 5.3° in the control group, with fewer outliers in the test group. The two groups showed no significant differences in the coronal femoral and tibial component angles or in the sagittal femoral and tibial component angles. The postoperative patient satisfaction was higher than 90% in both groups. The researchers concluded that TKA assisted by China’s domestic robot system could significantly improve the accuracy of prosthesis positioning, angle and lower limb alignment and gap balance, and the majority of patients were willing to undergo robot-assisted contralateral knee surgery (Table 1).

Professor Yuan Zhang from the Army Medical University gave a presentation on “Domestic Robot-Assisted TKA: Clinical Performance and Learning Curve”. He defined the LC as the time and practice required for a surgeon to reach a certain technical goal and a reflection of the correlation between the surgeon’s effort and the clinical outcome. Currently, LC at home and abroad is defined using the total operative time, anxiety level and the number of operations as criteria, and it does not yield an accurate result [33]. Given that the LC is associated with multiple perioperative factors, he classified it into four categories: operative time-based LC, the imaging index-based LC, the surgical trauma indicator-based LC and the postoperative functional recovery-based LC. He presented the results of a clinical trial of TKA assisted by the Yuanhua robot for examplification. The results showed that 6–8 cases fell into the operative time-based LC category. In terms of surgical trauma indicators, the average total blood loss of the test group and the control group were 650.1 mL and 751.6 mL, respectively; the hemoglobin levels of the two groups dropped by 21.1 g/L and 26.2 g/L, respectively; the erythrocyte sedimentation rate of the two groups increased by 2.02 mm/h and 3.53 mm/h 1 month after surgery, respectively; and the C-reactive protein of the two groups rose by 0.81 mg/L and 3.75 mg/L 1 month after surgery, respectively. The postoperative imaging evaluation showed that the lower limb joint line angles of the two groups were 178.53° and 177.26°, respectively (*P* < 005), and the postoperative slope of the tibial plateau were 3.59° and 4.44° in the two groups, respectively (*P* < 0.05), suggesting accuracy was higher in the test group than in the control group for restoring mechanical force lines and controlling the tibial slope. In terms of surgical trauma and imaging indicators, there was no LC for TKA. In terms of the postoperative function, the Knee Society Scores of the two groups increased by 53.2 and 50.2, respectively, 3 months after surgery (*P* < 0.05), and the HSS scores in the two groups increased by 15.4 and 12.1, respectively, 3 months after surgery (*P* < 0.05), suggesting that 14 cases were in the functional score-based (KSS, HSS) LC category. The two groups showed no significant difference in postoperative complications. The power of this study lies in the fact that it quantitatively assesses and accurately defines the LC for robot-assisted TKA, which can provide valuable support to help beginners avoid intraoperative risks, reduce the occurrence of complications and ensure surgical safety (Table 1).

**Table 1:** Summary of artificial intelligence assisted joint surgery in China in 2021

Studies	System	Origination	Main findings
Li *et al,* 2019	Digital twin	publication [3]	The Tang's classification divided fractures into anterior, posterior, lateral, and medial types. The majority of fracture lines (85.9%, 433/504) on the anterior maps were along the intertrochanteric line where the iliofemoral ligament was attached.
Kong *et al*, 2020	5G + Mako	publication [8]	First report on the application of 5G communication technology in the field of joint surgery. The increase of Harris scores of the two patients after surgery were 71 and 62, respectively.
Lei *et al*, 2019	MR + 3D	publication [10]	The first study to use MR combined with 3D printing technology in THA. The distance between the preoperative design rotation center and the postoperative rotation center was 5.5 mm. The postoperative anteversion angle was 30.67° and inclination was 43.16°, compared to preoperative design (anteversion angle 25° and inclination 40°).
Wu *et al*, 2019	5G + MR	publication [12]	The first 5G + MR-assisted remote consultation and screw fixation of a thoracic fracture in July 2019. The average depth for C1 and C2 pedicle screws were (29.82 ± 1.36) and (32.24 ± 1.21) mm on the left side, and (30.38 ± 0.95) and (31.42 ± 1.05) mm on the right side.
Guo *et al*, 2007	V-Xp Stryker	publication [13]	The acetabular abduction angle of computer navigation-assisted THA was in the range of 30°-54°, with an average of (40.6 ± 5.1) °, and an acetabular abduction angle of > 50° was observed only in 1 case; in patients who underwent conventional THA, the acetabular abduction angle was in the range of 28°-70°, with an average of (44.2 ± 8.7) °, and an acetabular abduction angle of > 50° was observed in 10 cases.
Zhang *et al*, 2011	BrainLab	publication [14]	Nine knee implants (28%) in the conventional group, compared with no knee implants in the computer-navigation group, deviated > 3° from the mechanical axis on the coronal plane. The coefficient variation of data in the conventional group was three times greater than that in the computer-navigation group.
Liu *et al*, 2020	StealthStation S7 (Medtronic)	publication [17]	The hidden blood loss in the patients underwent computer navigation-assisted TKA was significantly lower than in those who underwent conventional TKA (448.7 ± 300.7 mL *vs*. 688.6 ± 405.8 mL by GROSS method and 374.3 ± 360.8 mL *vs*. 667.2 ± 425.5 mL by HB-balance method).
Zhou *et al*, 2021	Mako	publication [18]	The Mako robot-assisted THA accuracy rates for implanting the acetabular cup into the Lewinnek and Callanan safety zones were 94.9 and 74.6%, compared to conventional THA (79.7 and 50.8%).
Li *et al*, 2021	Mako	publication [19]	The postoperative LLD of Mako robot-assisted THA and conventional THA were 2.3 mm and 6.7 mm (*P*<0.001).
Chai *et al*, 2020	Mako	publication [20]	The proportion of cases with the acetabular rotation center in the safety zone in the Mako robot-assisted group was 94.29% (*P* = 0.042), which was higher than conventional THA group (67.56%).
Chai *et al*, 2020	Mako	publication [21]	Robot-assisted surgery is suitable for complex THA, the postoperative Harris scores were 83/86 (left/right) for hip dysplasia, 87 for post-traumatic hip osteoarthritis and 62 (poor) for ankylosing spondylitis after robot-assisted surgery.
Fu *et al*, 2017	Mako	publication [25]	The recommended learning curve (LC) of Mako robot-assisted UKA was 8 cases.
Zhu *et al*, 2019	Mako	publication [27]	The increase of Knee Society clinical and functional scores were 37.2 and 29, and the range of motion of the knee joint increased 37.2° in Robotic-assisted UKA patients.
Wang *et al*, 2021	HURWA	publication [28]	No significant difference in the correction rate of HKA between robot surgery group and traditional surgery group (63.6% *vs*. 69.2%, *P* = 0.651).
Xia et al, 2021	Skywalker	publication [29]	100% of the absolute error of the HKA angles were within 3°; 90.32% of the postoperative lower limb alignment angles in 31 patients were close to 180° after the operation.
Chai *et al*, 2020	YUANHUA	publication [31]	The deviation between the planned cutting thickness before operation and the measured value after the operation was < 1 mm and that the error of the osteotomy angle was < 2° in the goat models.
Chai *et al*, 2020	YUANHUA	publication [32]	The postoperatively measured hip-knee-ankle angle was 177.1°–179.7°, the coronal femoral component angle was 87.9°–91.4°, and coronal tibial component angle was 87.3°–91.4°, with errors being within ±3° of the angles planned before operation in cadaver trials.
Chai W, 2021	Mako	conference speech (2021 ICRA)	A abnormal hip-spine relationship is possible in THA patients with dislocation due to unclear causes. An improved safety zone calculation method was established to provide accurate guidance for acetabular cup implantation.
Zhang XG, 2021	Mako	conference speech (2021 ICRA)	Among 64 Mako robot-assisted THA cases, 2 experienced loosening of the iliac assembly that caused registration failure; 1 case suffered iliac bone fracture; 3 cases had errors in acetabulum registration that caused the misalignment of the acetabular cup due to anteversion; and 1 case showed acetabular cup loosening.
Zhang Y, 2021	YUANHUA	conference speech (2021 ICRA)	Four types of “Learning Curve (LC)” was redefined in robotic-assisted TKA: LC in terms of operative measures was 6–8 cases; No LC exist in terms of radiologic results; LC in terms of postoperative function scores (KSS, HSS) was 14 cases; the terms of traumatic variables, such as blood loss, erythrocyte sedimentation rate, C-reactive protein, were less than conventional group (*P <* 0.05).
Zhang Y, 2019	StealthStat-ion S7 (Medtronic)	conference speech (2019 Chinese Knee Society)	In a clinical study of 14 knee OA with constitutional femoral varus, 7 patients underwent computer navigation-assisted TKA, none had pie-crust releasing of MCL or peeling off its posteromedial tibial attachment; compared to 4 cases of releasing of the superficial MCL and 3 cases of peeling off the tibial attachment in 7 patients of conventional TKA.
Zhou YX, 2021	Mako	conference speech (2021 ICRA)	An improved registration method of robot-assisted surgery was developed for acetabular revision. Among 45 cases of acetabular revision, the acetabular cup was within the Lewinnek safety zone in 43 cases and within the Callanan safety zone in 38 cases.
Zhou ZK, 2021	YUANHUA	conference speech (2021 ICRA)	The postoperative lower limb axis deviation ranges were 0–2° for the robot-assisted group and 0–5° for the conventional group; The mean posterior slope of the tibial component was 2.8° in the test group and 5.3° in the control group, with fewer outliers in the test group.

## Conclusion

AI-based precision, intelligentization and personalization represent the general trend in joint surgery. The success of multiple entities in independently developing domestic surgical robots in China showed that the deep integration of medicine, engineering and network has begun to take shape and that China will play an important role in AI-assisted medical care. However, robot-assisted surgery involves only robotic arm assistance at present, and the automation and intelligentization of this modality need to be further improved. Robots should be designed to use MRI data segmentation to achieve personalized algorithms for calculating cartilage thickness and soft tissue tension to match intraoperative fast scanning and registration capabilities, thereby transforming mechanical physical parameters to biomechanical characteristics and ultimately achieving the goal of individualized anatomical reconstruction.

Although there are few reports on the application of AI in shoulder, elbow and ankle surgeries in China, with the advancement AI-related techniques, its value in these surgeries will be shown, in terms of significantly improved accuracy and specificity of the operation, and further promoted digitalization and intelligentization of the surgical treatment.

## Data Availability

The datasets used and/or analyzed in the current study are available from the corresponding author on reasonable request.

## References

[1] Proceedings of 2005 IEEE Computational Systems Bioinformatics Conference. August 8-11, 2005. Stanford, California, USA. Proc IEEE Comput Syst Bioinform Conf. 2005:3–402. 17471682

[2] Konig IR, Fuchs O, Hansen G, von Mutius E, Kopp MV. What is precision medicine?. Eur Respir J. 2017;50(4):1700391. 10.1183/13993003.00391-2017. 10.1183/13993003.00391-2017 29051268

[3] Li J, Tang S, Zhang H, Li Z, Deng W, Zhao C (2019). Clustering of morphological fracture lines for identifying intertrochanteric fracture classification with Hausdorff distance-based K-means approach. Injury.

[4] Li M, Li ZR, Li JT, Lei MX, Su XY, Wang GQ (2019). Three-dimensional mapping of intertrochanteric fracture lines. Chin Med J.

[5] Maier-Hein L, Vedula SS, Speidel S, Navab N, Kikinis R, Park A (2017). Surgical data science for next-generation interventions. Nat Biomed Eng.

[6] Soffer S, Klang E, Shimon O, Nachmias N, Eliakim R, Ben-Horin S (2020). Deep learning for wireless capsule endoscopy: a systematic review and meta-analysis. Gastrointest Endosc.

[7] Teber D, Engels C, Maier-Hein L, Ayala L, Onogur S, Seitel A (2020). Surgery 4.0-are we ready?. Urologe A.

[8] Kong XP, Fu J, Chen JY, Chai W, Wu D, Li ZF (2020). 5G communication technology remotely guides two cases of robot-assisted total hip arthroplasty. Chin J Reparative Reconstr Surg.

[9] Hu HZ, Feng XB, Shao ZW, Xie M, Xu S, Wu XH (2019). Application and prospect of mixed reality technology in medical field. Curr Med Sci.

[10] Lei PF, Su SL, Kong LY, Wang CG, Zhong D, Hu YH (2019). Mixed reality combined with three-dimensional printing technology in total hip arthroplasty: an updated review with a preliminary case presentation. Orthop Surg.

[11] Lacy AM, Bravo R, Otero-Pineiro AM, Pena R, De Lacy FB, Menchaca R (2019). 5G-assisted telementored surgery. Br J Surg.

[12] Wu X, Liu R, Xu S, Yang C, Yang S, Shao Z (2019). Feasibility of mixed reality-based intraoperative three-dimensional image-guided navigation for atlanto-axial pedicle screw placement. Proc Inst Mech Eng H.

[13] Guo XZ, Dou BX, Liu Q, Huang Y, Zhou YX (2007). Comparison of the acetabular orientation after minimally-invasive total hip arthroplasty with and without computer-navigation: a clinical report of 106 hip in 87 patients. Natl Med J China.

[14] Zhang GQ, Chen JY, Chai W, Liu M, Wang Y (2011). Comparison between computer-assisted-navigation and conventional total knee arthroplasties in patients undergoing simultaneous bilateral procedures: a randomized clinical trial. J Bone Joint Surg Am.

[15] Hampp EL, Chughtai M, Scholl LY, Sodhi N, Bhowmik-Stoker M, Jacofsky DJ (2019). Robotic-arm assisted total knee arthroplasty demonstrated greater accuracy and precision to plan compared with manual techniques. J Knee Surg.

[16] Zhang Y, Gu WH (2020). Artificial intelligence in knee surgery: status and prospect. J Trauma Surg.

[17] Liu ZY, Zhang J, Li J, He K, Zhang YM, Zhang Y (2020). The role of continuous optimization program in damage control of perioperative blood loss during primary total knee arthroplasty. J Trauma Surg.

[18] Zhou Y, Shao H, Huang Y, Deng W, Yang D, Bian T (2021). Does robotic assisted technology improve the accuracy of acetabular component positioning in patients with DDH?. J Orthop Surg (Hong Kong).

[19] Li JC, Li M, Ji QB, Sun JY, Zheng QY, Zhang GQ (2021). Comparison of lower limb length difference between robot-assisted and traditional methods of total hip arthroplasty. Chin J Orthop.

[20] Chai W, Kong X, Yang M, Puah KL, Tang P, Chen J (2020). Robot-assisted total hip arthroplasty for arthrodesed hips. Ther Clin Risk Manag.

[21] Chai W, Guo RW, Puah KL, Jerabek S, Chen JY, Tang PF (2020). Use of robotic-arm assisted technique in complex primary total hip arthroplasty. Orthop Surg.

[22] DelSole EM, Vigdorchik JM, Schwarzkopf R, Errico TJ, Buckland AJ (2017). Total hip arthroplasty in the spinal deformity population: does degree of sagittal deformity affect rates of safe zone placement, instability, or revision?. J Arthroplast.

[23] Ike H, Dorr LD, Trasolini N, Stefl M, McKnight B, Heckmann N (2018). Spine-pelvis-hip relationship in the functioning of a total hip replacement. J Bone Joint Surg Am.

[24] Zhang J, Huang Y, Zhou B, Zhou Y (2019). Mid-term follow-up of acetabular revision arthroplasty using jumbo cups. Orthop Surg.

[25] Fu J, Chai W, Ni M, Li X, Liu K, Kong XP (2019). Learning curve difference between robotic-assisted and conventional Oxford unicompartmental knee arthroplasties. Chin J Joint Surg (Electronic Edition).

[26] Di Martino A, Bordini B, Barile F, Ancarani C, Digennaro V, Faldini C (2021). Unicompartmental knee arthroplasty has higher revisions than total knee arthroplasty at long term follow-up: a registry study on 6453 prostheses. Knee Surg Sports Traumatol Arthrosc.

[27] Zhu KC, Wang QJ, Chen YS, Shen H, Peng XC, Zhang XL (2019). Short-term clinical outcomes of robotic-assisted unicompartmentalknee arthroplasty. Chin J Joint Surg (Electronic Edition).

[28] Wang W, Chen X, Fan Y, Lin J (2021). A clinical study on robotic-assisted total knee arthroplasty for severe knee osteoarthritis. Chin J Bone Joint Surg.

[29] Xia R, Zhai Z, Zhang J, Yu D, Wang L, Mao Y (2021). Verification and clinical translation of a newly designed “Skywalker” robot for total knee arthroplasty: a prospective clinical study. J Orthop Translat.

[30] Xia RZ, Tong ZC, Zhang JW (2021). Early clinical study of domestic “Skywalker” surgical robot knee arthroplasty. J Pract Orthop.

[31] Chai W, Xie J, Zang XG, Yan TF, Zhao YL, He CA (2020). Animal experimental study on domestic robot-assisted total knee arthroplasty. Chin J Reparative Reconstr Surg.

[32] Chai W, Xie J, Zang XG, He C, Yan TF, Liu L (2021). A cadaveric experimental study on domestic robot-assisted total knee arthroplasty. Chin J Reparative Reconstr Surg.

[33] Kayani B, Konan S, Huq SS, Tahmassebi J, Haddad FS (2019). Robotic-arm assisted total knee arthroplasty has a learning curve of seven cases for integration into the surgical workflow but no learning curve effect for accuracy of implant positioning. Knee Surg Sports Traumatol Arthrosc.

